# A Fluorescent Chromatophore Changes the Level of Fluorescence in a Reef Fish

**DOI:** 10.1371/journal.pone.0037913

**Published:** 2012-06-06

**Authors:** Matthias F. Wucherer, Nico K. Michiels

**Affiliations:** Animal Evolutionary Ecology, University of Tübingen, Tübingen, Germany; The Australian National University, Australia

## Abstract

Body coloration plays a major role in fish ecology and is predominantly generated using two principles: a) absorbance combined with reflection of the incoming light in pigment colors and b) scatter, refraction, diffraction and interference in structural colors. Poikilotherms, and especially fishes possess several cell types, so-called chromatophores, which employ either of these principles. Together, they generate the dynamic, multi-color patterns used in communication and camouflage. Several chromatophore types possess motile organelles, which enable rapid changes in coloration. Recently, we described red fluorescence in a number of marine fish and argued that it may be used for private communication in an environment devoid of red. Here, we describe the discovery of a chromatophore in fishes that regulates the distribution of fluorescent pigments in parts of the skin. These cells have a dendritic shape and contain motile fluorescent particles. We show experimentally that the fluorescent particles can be aggregated or dispersed through hormonal and nervous control. This is the first description of a stable and natural cytoskeleton-related fluorescence control mechanism in vertebrate cells. Its nervous control supports suggestions that fluorescence could act as a context-dependent signal in some marine fish species and encourages further research in this field. The fluorescent substance is stable under different chemical conditions and shows no discernible bleaching under strong, constant illumination.

## Introduction

Reef fish are colorful for many ecological reasons [1] and therefore it is not surprising that the resultant color patterns must comply with many requirements. Two functions of body coloration seem to be contradictory: In intraspecific communication such as courtship and territoriality, colorful displays often serve as a presentation of signals [2], [3]. In aposematic predator-prey communication, animals use their body coloration to signal inedibility [4]. In both cases colors are used to become as conspicuous as possible. For predator avoidance or prey capture, however, it is more important for an animal to be cryptic and well camouflaged [5], [6]. One evolutionary solution to these opposing selective forces is adaptive body coloration [4], [5]. This is achieved through specialized cells in the skin that vary their appearance from clearly visible to almost invisible [7]. In poikilotherms, particularly in fishes, a broad variety of these so-called chromatophores has evolved. Each of these is specialized to display a certain color by means of different color-generating structures [8].

Within these structures, two types of colors are used: pigment colors and structural colors [9]. Pigments generate color impressions by (partial) absorption and complementary (partial) reflection of the incoming light. Structural colors use crystalline structures to generate colors by scattering, thin-film interference and diffraction [9].

Chromatophores use either of these mechanisms to generate a specific color appearance [9] (Table 1). Most pigment-containing chromatophores (melanophores, erythrophores, xanthophores and cyanophores) are not only specialized in producing and storing, but also in translocating their numerous pigment particles [8], [10]. They increase color visibility by radial particle dispersal or reduce it by concentric particle aggregation. In contrast to these pigment-based mechanisms, iridophores are chromatophores, which use refractive guanine platelets to create a silvery appearance or to generate structural colors (like most blue hues in fishes) [11]. Iridophores can vary the configuration of platelet-units to alter hue or brightness [12]. Both mechanisms show that chromatophores are the fundamental system for physiological control over body coloration [3], [13]. The ability to quickly change color solves the dilemma between having to be conspicuous for communication and inconspicuous for camouflage through context-dependent plasticity [8].

**Table 1:** Different chromatophores (according to [9], modified).

Appearance	Chromatophore	Physical Principle	Color Change Mechanism
brown/black	melanophore	absorption	pigment translocation
yellow/orange	xanthophore	absorption/reflection	
red	erythrophore	absorption/reflection	
blue	cyanophore	absorption/reflection	
blue	iridophore	thin film interference (structural color)	change in configuration of guanine platelet units
silver	iridophore	refraction	
white	leukophore	scatter	–

The bright colors of coral reef fishes have already been the subject of many studies (e.g. [5], [14]). Despite this, and due to the progress of objective measurement techniques, there are many aspects that have been discovered only recently [16]. For instance, the fact that damselfish use ultraviolet communication has been overlooked before due to its invisibility to the human observer [17]. The presence and expression of genes encoding UV-sensitive opsins also suggests the importance of UV communication in a number of fish species [18], [19].

UV-radiation might not be the only part of the light spectrum that is used more in visual communication than previously assumed. For instance, red displays cannot be generated by reflection in depths below 10 to 20 m, because water quickly attenuates the longer wavelengths (>600 nm) of the incoming sunlight [20]. As a consequence, pigments cannot reflect red and will appear grey or black in most of the euphotic zone of many marine environments. It is therefore assumed that for fishes in these depths blue and yellow hues are more important than red (see [13], [21] and references therein). This implicitly assumes that all long (“red”) wavelengths in the environment stem from the sun exclusively. In deep-sea fishes, however, red bioluminescence acts as a local source of red light in the dark (e.g. [22]).

Red fluorescent pigments can absorb ambient blue-green light and re-emit the light energy as photons at a longer wavelength. Therefore, fluorescence could be a suitable mechanism to generate a local source of red light in otherwise red-depleted, euphotic marine habitats below 10 m [23].

**Figure 1:**
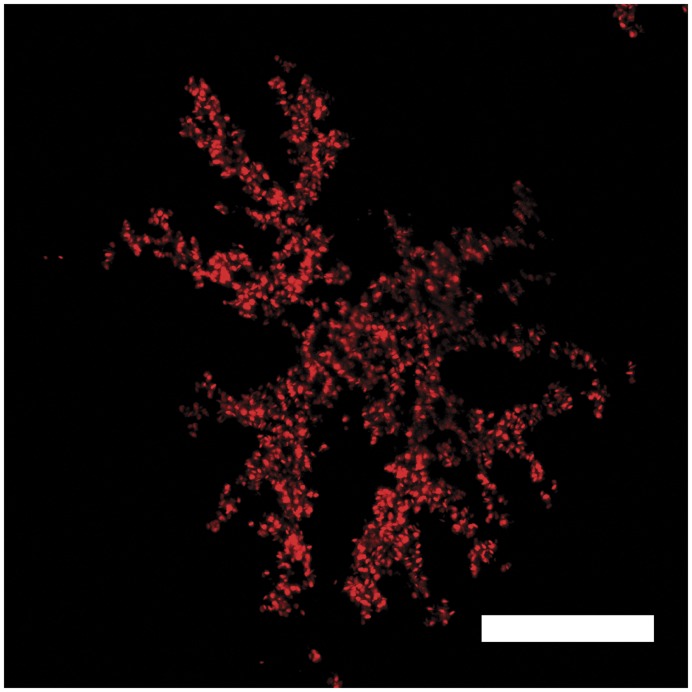
Morphology of a fluorescent chromatophore. Confocal Laser scanning microscopy, excitation 510 nm. Fluorosomes are the only visible structures within the fluorescent cell, but in the dispersed state they show the cell outline as they fill the cytoplasm entirely. Scale bar  = 30 µm.

A strong indicator that such red fluorescence could be used as a signal would be the observation of active color modulation as shown by chromatophores [8]. Here, we describe the discovery of a chromatophore with motile fluorescent organelles in the skin of the pygmy coral reef goby *Eviota pellucida* (Larson 1976). This species shows red fluorescence mainly in two lateral stripes, the eye rings and all but the pectoral fins [23]. Fluorescent cells are mainly located in the interradial membranes of the fins and are invisible under brightfield and darkfield light microscopy. Their shape is dendritic and flat in the plane of the interradial membrane (Figure 1).

We characterize these fluorescent cells and test whether they are controlled by neurons and/or hormones. If so, their fluorescence could be part of an adaptive body coloration system and may indicate the existence of communication on the long-wavelength side of the visible spectrum in marine fishes. We compare the fluorescent cells to other chromatophores on the basis of well-described characteristic features of melanophores [24]. The intrinsic neurotransmitter involved in this brain-controlled change of darkness of melanophores is noradrenaline [25], [26].

In addition to rapid neuronal changes, hormones can also influence body coloration. The Melanosome Concentrating Hormone (MCH) and the Melanophore Stimulating Hormone (MSH) are well known endocrine mediators regulating the appearance of melanophores [27], [28].

We tested the following hypotheses to examine if neurons, neurotransmitters and hormones are involved in a controlled change of fluorescent color:

1. Stimulation of efferent neurons leads to an aggregation of fluorescent pigments, thus reducing the exposure of the fluorescent pigments.
2. External noradrenaline stimulates the same response by mimicking synaptic release of this neurotransmitter.
3. Application of MCH leads to aggregation and α-MSH makes the cells disperse their fluorescent pigments.

## Results

**Figure 2:**
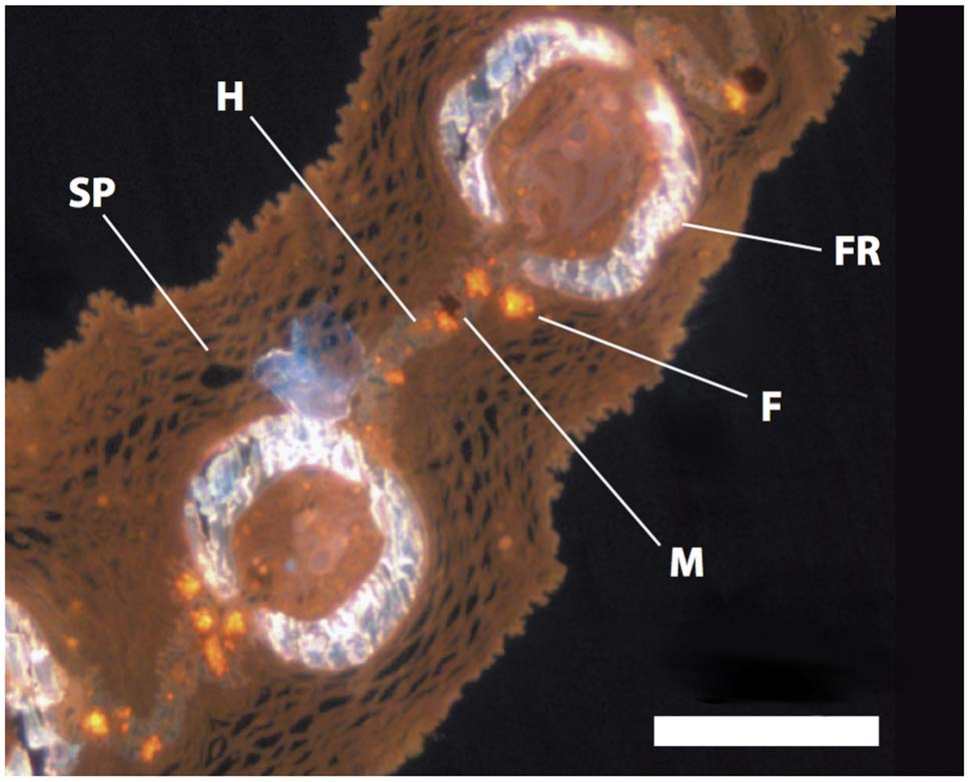
Position of fluorescent chromatophores in situ. Histological cross-section of first dorsal fin of *E. pellucida* (fluorescence microscopy): M = Melanophore, F = Fluorescent Chromatophore, FR = Fin Ray, H = Hypodermis, SP = Stratum spongiosum. Scale bar  = 500 µm.

**Figure 3:**
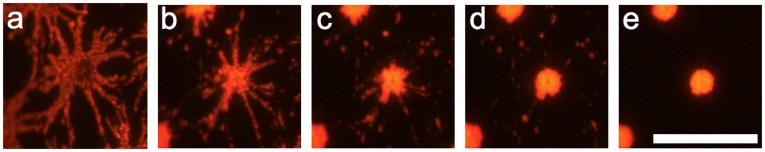
Spectra of excitation and emission of the fluorescent pigment in fluorescent chromatophores. Fluorescence has a maximum at around 595 nm (dashed line) with an optimal excitation wavelength around 500 nm (solid line).

**Figure 4:**
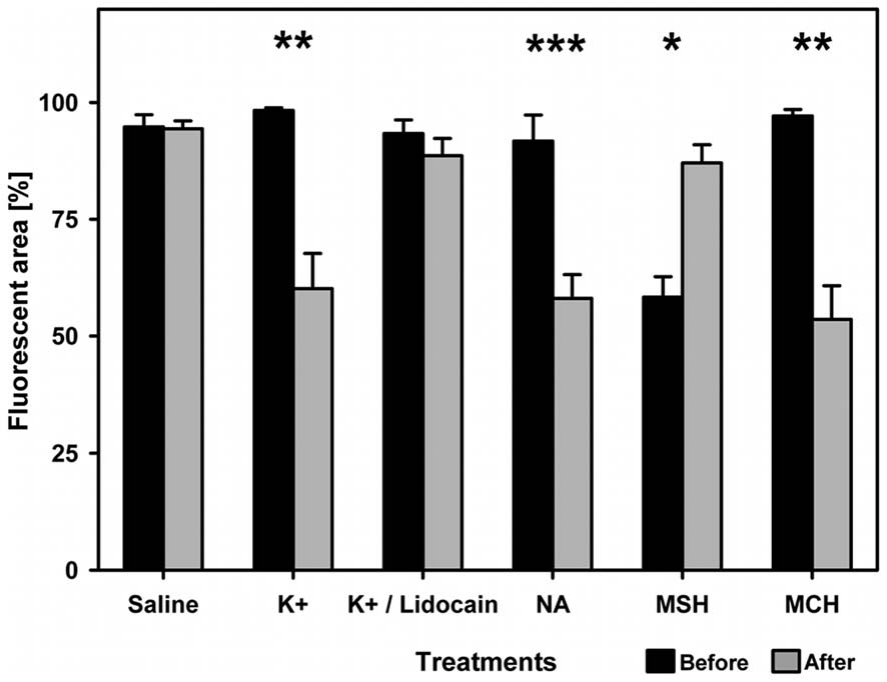
Distribution of erythrophores, melanophores and fluorescent chromatophores in the interradial membrane of a dorsal fin of *E. pellucida*. **a**) Erythrophores (red) and melanophores (black) are visible in bright field microscopy. **b**) Fluorescent chromatophores appear in fluorescence microscopy. **c**) Overlay of a) and b). Note that erythrophores, melanophores and fluorescent chromatophores are spatially distributed and can be distinguished. Scale bar  = 400 µm.

Histological sections show that fluorescent cells are present exclusively below the epidermis between mineralized fin rays together with other chromatophores (Figure 2). These cells are on average 85 µm in diameter (±16.8 µm SD, n = 12) and their intracellular fluorosomes measure 1.3 µm (±0.2 µm SD, n = 10). Spectrometric measurements show that the red coloration produced by these cells is pure fluorescence with no reflectance. Optimal excitation occurs at around 500 nm, whereas most red light is emitted at 595 nm (Figure 3). Figure 4 shows red erythrophores and melanophores in bright field microscopy. The fluorescent cells are almost invisible under these conditions. In fluorescence microscopy, however, they appear, but spatially separated from other chromatophores.

**Figure 5:**
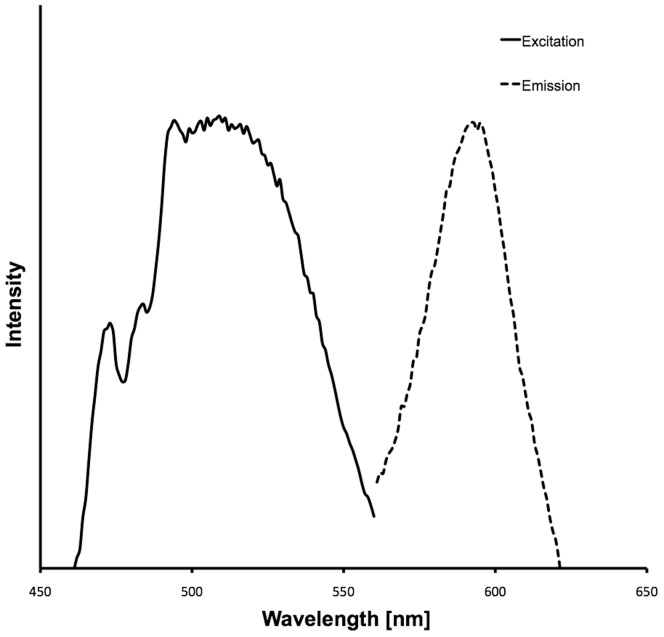
Aggregation of fluorescence. Classified according to melanophore index 5 (a) through melanophore index 1 (e, respectively). In a completely dispersed state (a) the nucleus becomes visible as there is only little cytoplasm one the apical and basal side of the nucleus in these flat cells. In the aggregated state (e), the nucleus is tightly packed with fluorosomes. Scale bar  = 100 µm.

**Figure 6:**
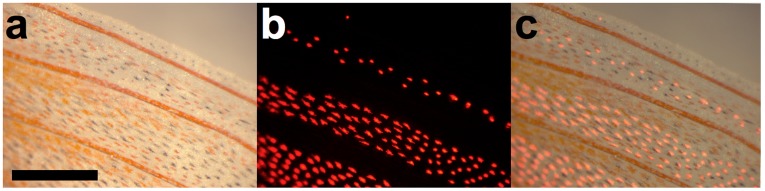
Results of cell manipulation. Bars show results of neuronal and hormonal manipulation in terms of normalized fluorescent area in interradial membranes of *E. pellucida* before (white bars) and after treatment (shaded bars, respectively). Neuronal K^+^ stimulation significantly decreased fluorescent area (paired t-test, t = 10.5, df = 5, p<0.005). The Lidocain-treatment effectively inhibited this effect (paired t-test, t = 1.86, df = 4, p>0.13). Neurotransmitter-induced aggregation of fluorosomes (NA) was highly significant (paired t-test, t = 4.67, df = 9, p<0.001). Aggregation induced by MCH was significant (paired t-test, t = 5.19, df = 3, p<0.013) as well as α–MSH significantly induced dispersal in pre-aggregated cells (paired Wilcoxon, Z = 10.5, df = 5, p = 0.03). Bars include standard errors.

In all three types of manipulation, the fluorescent cells have translocated their fluorosomes either towards the nucleus or away from it (Figures 5 and 6). There was a significant reduction of fluorescent area after (neuronal) K^+^ stimulation (paired t-test, t = 10.5, df = 5, p<0.005). This change was effectively inhibited by the addition of Lidocain to the high K^+^-solution (paired t-test, t = 1.86, df = 4, p>0.13). The neurotransmitter (NA) treatment also resulted in a highly significant aggregation of fluorosomes (paired t-test, t = 4.67, df = 9, p<0.001).

In the hormonal treatments the fluorescent chromatophores also act analogous to melanophores. Stimulated by MCH, the cells significantly aggregated fluorescent pigments (paired t-test, t = 5.19, df = 3, p<0.013), whereas α–MSH provoked dispersal in previously aggregated cells (paired Wilcoxon, Z = 10.5, df = 5, p = 0.03).

We did not find changes in fluorescence in the saline control group (paired Wilcoxon, Z = −0.5, n = 6, p>0.1.).

Single fluorosomes were translocated towards the nucleus at a speed of 0.49 µm/s (±0.09 SD, n = 10). For timelapse-recordings of fluorosome-translocation see Videos S1 and S2. Total time for the KCl-induced, complete aggregation was on average 523 s (±98 SD, n = 10).

## Discussion

The fluorescent cells of *E. pellucida* are dendritic in shape, and with a diameter of approximately 85 µm they are within the range of other teleost chromatophores (compare [24], [29]). They aggregated their fluorosomes significantly in the KCl-, neurotransmitter (NA)-, and melanosome concentrating hormone treatment (MCH). Melanophore Stimulating Hormone had the opposite effect: pre-aggregated cells dispersed their fluorescent particles after application. In general, the cells have shown their capacity to increase and decrease the visible area of fluorescent pigments. These results show that these cells share central functions with (true) pigment chromatophores.

Some chromatophore types, mainly melanophores, have been shown to translocate pigment particles (melanosomes) after stimulation of neurons by increased K^+^ levels. A sophisticated system of Dynein- and Kinesin-motorproteins carries melanosomes along the cytoskeleton from dendrites towards the perikaryon and vice versa [30], [31]. The results of the treatment with elevated K^+^ concentrations show significant aggregation of fluorosomes, which confirmed the prediction that pigments can be aggregated by neuronal stimuli. We conclude that it is likely that fluorescent cells are innervated in the same way as melanophores are.

To exclude direct action of K^+^ on fluorescent chromatophores, sodium channels of neurons were selectively blocked using Lidocain and therefore no more action potentials could be released. In this treatment, elevated K^+^ did not release a response in fluorescent chromatophores. From this follows that translocation of fluorescent particles depends at least in part on neuronal sodium channels and that this mechanism is under neuronal control. If K^+^ had an effect other than eliciting action potentials in these neurons, it should not have been possible to prevent cell response by blocking sodium channels.

The fluorescent chromatophores also clearly responded to experimental application of NA, the neurotransmitter in neuron-melanophore-connections. This suggests the existence of a connection between neurons and fluorescent cells similar to other chromatophores.

From the three experimental treatments, we conclude that these fluorescent cells are controlled by the nervous system just as other chromatophores (compare [8]). Rapid nervous control suggests that the fish might use this fluorescence for a purpose that requires rapid change in fluorescent color.

In addition, α–MSH and MCH, both hormones known for their melanophore-modulating properties, showed significant effects on the fluorescent cells. Fluorescent particles dispersed when stimulated with α–MSH and aggregated when stimulated with MCH. Again, this is similar to the response known from melanophores. In general, hormonal responses are much slower than those resulting from neuronal action. Melanophore responses to α–MSH and MCH are used for color changes during diurnal rhythm [32], stress response [3], or background adaptation [33] but have been shown to also modulate sensitivity towards neuronal input [34]. The same responses to hormonal stimuli as in melanophores suggest that these cells could be used and modulated at least for one of the purposes described above.

The results suggest that these cells could potentially be used for adaptive body coloration and its functions as is the case for other chromatophores. Behavioral studies are needed to examine if fluorescent fishes actually use their fluorescence control mechanisms, e.g. for signaling or camouflage.

Morphological traits, which are typical for motile chromatophores such as size, dendritic shape and pigment translocation, suggest a close relationship to melanophores. However, in the cells investigated, we did not find dark pigments, which define certain chromatophores as melanophores. On the other hand, the fact that these cells fluoresce in red could imply a relationship to erythrophores, which are responsible for red appearance under broad-spectrum light conditions. In addition, some pigments in erythrophores and xanthophores, namely pterins and pteridines, can also weakly fluoresce [29]. In contrast to red reflective pigments of erythrophores, red fluorosomes are barely visible under broad spectral light, indicating that their chromaticity is based on red fluorescence only. In contrast to fluorescence as a side effect under UV-excitation in inbred guppy strains [29], red fluorescence in the cells investigated here occurs in natural populations of *E. pellucida* under natural illumination regimes [23].

It is likely that red fluorescence in reef fish (including *E. pellucida*) is associated with guanine crystals [23], which are also the optically active components of iridophores. It could be argued that the chromatophores described here contain fluorescence-associated guanine and that they could be iridophores, which show fluorescence as an additional feature. Iridophores respond to neuronal and/or hormonal stimuli by changing the angle to incoming light and therefore also the refractive properties of guanine platelets [9]. This mechanism, however, is fundamentally different from the pigment translocation observed here in the fluorescent chromatophores. Moreover, iridophore response to various stimuli is reciprocal to that of other chromatophores [8], whereas fluorescent chromatophores react exactly like pigment chromatophores.

In all responses investigated here, fluorescent chromatophores responded the same way as melanophores and other non-iridophore chromatophores. The speed of fluorosome aggregation is 0.49 µm/s (±0.09 SD) and therefore very similar to translocation of melanosomes in other teleost species, which varies between 0.5–1.5 µm/s [24]. In contrast, motility in iridophores is much slower [8], [24]. Hence, although both cell types may contain guanine, it can be excluded that fluorescent chromatophores are modified iridophores.

For these reasons, we suggest that these fluorescent chromatophores could be a separate, novel type of chromatophore related to melanophores. It is likely that many fluorescent fish species possess these cells. In addition to being a new type of chromatophore, our findings have two other important implications: First, fluorescence is naturally bound to the cytoskeleton (presumably via adaptor proteins), as is the case in melanophores [30], [35] and it is stable under strong, constant illumination. These cells may provide insights into intracellular transport processes especially in combination with differently colored fluorescent markers. Second, due to both rapid (nervous control) and long-term (hormonal control) modulation of fluorescence, our findings could potentially contribute to a new field of fish communication on the longer-wavelengths side of the visible spectrum even in environments devoid of the color red.

## Methods

Thirteen specimens of *E. pellucida* were obtained from an ornamental fish trader (Mrutzek Meeresaquaristik, Ritterhude, Germany). This species reaches a total length of 3 cm and lives on coral rubble in depths of up to 20 m. It is known to fluoresce in red with an emission peak at about 600 nm in several parts of the body [23]. Among other structures, it also possesses fluorescent dendritic cells in the inter-radial membranes of all but the pectoral fins. Fish were kept in marine aquaria before experiments started (salinity 34, temperature 26°C, pH 8.2). Fish were decapitated for the experiment and fins were cut off and stored in a physiological saline solution with the following composition: (in mM) NaCl 125.3, KCl 2.7, CaCl_2_ 1.8, MgCl_2_ 1.8, D−(+)-Glucose 5.6, Tris-HCl 5.0, pH 7.2. Single fins were put in a microscopic flow chamber (two microscopy slides divided by a 0.25 mm polypropylene-gasket) in the same saline.

### Then the Solution was Replaced According to the Three Tested Hypotheses

1. Saline with elevated K^+^ was given to stimulate action potentials in neurons (in mM: NaCl 78, KCl 50, CaCl_2_ 1.8, MgCl_2_ 1.8, D(+)-Glucose 5.6, Tris-HCl 5.0, pH 7.2; the total ion concentration was kept constant relative to the physiological solution). In an additional treatment, we also applied elevated K^+^-saline, but containing 100 µM Lidocain to exclude direct effects of K^+^ on the fluorescent cells. Lidocain is a specific blocker of neuronal sodium channels and prevents action potentials.
2. For the neurotransmitter-treatment, 2.5 µM of noradrenaline (NA) was applied. NA is the intrinsic neurotransmitter in fish chromatophores [25], [26].
3. For the hormonal treatments, α–MSH and MCH were diluted in saline to 1 µM.

### A Treatment with Plain Physiological Solution as Described above was Used as a Control

The chambers were put under a fluorescence microscope (Leica DM5000B, Leica Microsystems, Germany) and excited with monochromatic light, turquoise to the human eye (lowpass filter at 495 nm, Leica Microsystems, Germany). Directly after replacement of the initial saline every five seconds a still image was taken through an optical long-pass filter which only allows wavelengths from 590–660 nm to pass, thus cutting out the excitation light while allowing only the fluorescence to pass. Digital images were imported into ImageJ (V.10.2, J. Rasband, NIH, USA), set to 8-bit grayscale and inverted. A threshold was set to distinguish between fluorescent and non-fluorescent areas. Then the fluorescent area of every single image was determined by counting pixels. Data were normalized to full dispersal of fluorescent bodies as 100 %. For statistical analysis, timestamps and relative dispersal were entered into JMP (V.9 SAS, USA) and means were compared using pairwise tests (before and after treatment). Figure 6 was plotted using SigmaPlot (V.11, Systat, USA). On the basis of the melanophore index, which defines stages of pigment organelle dispersal in melanophores [36], 100 % dispersal was considered as a state similar to melanophore index 5. The aggregation speed of 5 fluorosomes on their way towards the nucleus was determined for each of 5 fluorescent chromatophores from 10 fish (n = 10) by measuring the distance left behind between still images with a known time difference using microscope-specific distance calibration in ImageJ. Aggregation time was calculated from the same data, whereby time was measured from start of the experiment to the latest observable aggregation movement.

Optimal excitation wavelengths and fluorescent emission wavelengths were measured from a fresh fin using a spectrofluorometer (QuantaMaster QM-40, Photomed, Germany).

To localize the fluorescent cells in situ, whole fins of *E. pellucida* were fixed in caco-buffer (0.1 M sodium cacodylate trihydrate, pH 7.6) containing 0.2 % glutardialdehyde for 2 weeks at 4°C. After dehydration in five steps of increasing ethanol dilutions from 70 % to 100 %, they were embedded in histological resin (EPON). Cross sections of 2.5 µm were cut, mounted on microscopy slides and examined without any staining under the fluorescence microscope mentioned above.

## Supporting Information

Video S1 **Timelapse recording of fluorophores in tissue aggregating after K^+^ stimulation.** Scale bar  = 100 µm, acceleration factor 1∶35.
(MOV)

Video S2 **Timelapse recording of a single fluorophore showing aggregation of fluorescent intracellular particles.** Scale bar  = 100 µm, acceleration factor 1∶35.
(MOV)
